# Predictors for pathologically confirmed aortitis after resection of the ascending aorta: A 12-year Danish nationwide population-based cross-sectional study

**DOI:** 10.1186/ar3360

**Published:** 2011-06-15

**Authors:** Jean Schmidt, Kaare Sunesen, Jette B Kornum, Pierre Duhaut, Reimar W Thomsen

**Affiliations:** 1 Department of Clinical Epidemiology, Aarhus University Hospital, Aalborg Hospital Science and Innovation Center, Sdr. Skovvej 15, DK-9000 Aalborg, Denmark; 2 Department of Internal Medicine and RECIF, Amiens University Hospital, place Victor Pauchet, 80054, Amiens, Cedex 1, France

## Abstract

**Introduction:**

Assessing the prevalence of, and predictors for, pathologically-confirmed inflammation of the aorta in Denmark, using a nationwide population-based study design.

**Methods:**

We identified all adults with first-time surgery on the ascending aorta between January 1, 1997 and March 1, 2009 in Denmark. Presence of aortic inflammation was ascertained through linkage to a nationwide pathology registry. We used logistic regression to compute prevalence odds ratios (ORs) for sex, age at surgery, cardiovascular risk factors, cancer, connective tissue disease, and infectious diseases associated with the presence of aortitis.

**Results:**

A total of 1,210 adults underwent resection of the ascending aorta, of who 610 (50.4%) had tissue submitted for pathological examination. Aortitis was found in 37 (6.1%) patients whose tissue was examined. Ten of the 37 patients were diagnosed with conditions known to be associated with aortitis or aortic aneurysm: five patients with temporal arteritis, one with Crohn's disease, one with rheumatoid arthritis, one with systemic lupus erythematosus, one with infectious aortitis, and one with Marfan's disease. Twenty-seven patients had idiopathic aortitis. Predictors of aortitis included history of connective tissue disease (adjusted OR 4.7, 95% confidence interval (CI) 1.6, 13.6), diabetes (OR 5.2, 95% CI 0.9, 29.7), advanced age (> 67 years OR 2.5, 95% CI 0.8, 7.6), and aortic valve pathology (OR 2.3, 95% CI 1.1, 4.9).

**Conclusions:**

Aortitis was present in 6.1% of adults in Denmark who had pathological examination after resection of the ascending aorta. Predictors of inflammation included connective tissue disease, diabetes, advanced age, and aortic valve pathology.

## Introduction

Aortitis is defined as inflammation of the aortic wall [1]. Numerous medical conditions have been associated with a risk of aortitis, but data from a large population-based study of aortitis risk factors are lacking. In spite of the rarity of infection, this possibility should be considered first as a cause of aortitis, because of the severity of the condition and the specificity of required treatment [2-5]. Next, inflammatory diseases should be considered, as aortitis may be a component of inflammatory diseases such as temporal arteritis [6] and Takayasu arteritis [7]. Although other diseases also have been associated with aortitis [8-15], available evidence is based mainly on case reports, and a large proportion of aortitis cases may be idiopathic. Idiopathic aortitis seems to affect particularly the ascending thoracic aorta, and is often diagnosed unexpectedly on the basis of pathological samples taken during surgery for aneurysm or dissection [1]. In previous series of pathologic examinations of tissue from patients with non-infectious thoracic aortitis, the two most commonly reported diagnoses were idiopathic aortitis and aortitis associated with temporal arteritis [16].

Potential life-threatening complications such as aortic aneurysm and dissection and the need for disease-specific treatment [17]make aortitis important to diagnose. Also, the presence of aortitis worsens the prognosis of patients undergoing aortic surgery [18,19]. Few data are available concerning risk factors for this condition [20]. A recent study focused on classical cardiovascular risk factors, but the pathophysiology of aortitis remains unclear and malignancies, infectious diseases, and other diseases could be associated with aortitis [21-27]. Previous studies on the epidemiology of aortitis had several limitations, including recruitment bias in specialized surgical centers [16,28,29] and an unknown proportion of patients whose tissue was sent to the pathology department for examination [29]. No previously published studies were population-based.

We used a nationwide registry that included all hospitalizations for surgery on the thoracic ascending aorta, in order to study the prevalence of aortitis among surgical patients over a 12-year period. We examined the association between classical cardiovascular risk factors (age, sex, diabetes, and hypertension), major comorbidities (ischemic heart disease, cerebrovascular diseases, connective tissue diseases, cancer, peripheral vascular disease, renal diseases, and infections), and the risk of thoracic aortitis documented through pathologic examination. Also, we determined the proportion of aortitis cases that were idiopathic.

## Materials and methods

### Setting and study population

This cross-sectional study was conducted in Denmark, with a population of 5,489,022 as of 1 July, 2008 (Statistics Denmark). The Danish National Health Service provides free access to tax-supported health care (primary care and hospital care) [30]. A unique civil personal registration number assigned to each Danish citizen at birth, which is included in all health databases, allowed us to link the different databases accurately.

We identified all patients hospitalized between 1 January, 1997 and 1 March, 2009 for first-time surgery of the thoracic ascending aorta (including resection of the aorta during the procedure) from the Danish National Patient Registry (DNPR). The registry covers all patients admitted to Danish non-psychiatric hospitals since 1977 and all patients treated in emergency rooms and outpatient clinics since 1995. Its data include date of admission, date of surgery, date of discharge, surgical procedures, and diagnoses. The surgical procedure codes relevant to our study were Nordic Medico-Statistical Committee (NOMESCO) classification of surgical procedure codes [31] corresponding to surgery on the ascending part of the aorta (NOMESCO codes: FCA50-70). This classification system has been used since 1996 in Denmark. Patients aged under 15 years at the time of surgery were excluded from the analysis.

### Aortitis

Among patients undergoing surgery on the ascending part of the aorta, we identified those whose tissue was submitted for pathologic examination through linkage with the National Pathology Registry. This registry contains data on all pathologic examinations performed in Denmark since 1 January, 1997, using the systematized nomenclature of medicine (SNOMED) codes [32]. This nomenclature allows for identification of the organ (i.e., code T42000-T42400 for the ascending aorta), and the diagnosis yielded by the pathologic examination (i.e., codes M4000-M47150 for inflammation, in the case of our study).

### Aortitis risk factors

For each eligible patient, a complete hospitalization history including major medical diagnoses and comorbidities was available through linkage to the DNPR. Diagnoses included in the DNPR were coded by physicians according to the *International Classification of Diseases *(ICD), 8^th ^revision until the end of 1993, and 10^th ^revision afterwards.

For each patient, we also recorded gender, age at surgery, and the surgical center performing the operation. Only five hospital departments in Denmark (Rigshospitalet Copenhagen, Gentofte Hospital Copenhagen, Odense Hospital, Aarhus Hospital, and Aalborg Hospital) perform surgery on the aorta.

Potential risk factors for aortitis were extracted from the DNPR at discharge following surgery. As well, potential risk factors recorded during hospital stays prior to surgery were collected. These included diabetes mellitus (type I or II), chronic or acute ischemic heart disease, hypertension, cerebrovascular diseases (intracerebral hemorrhage, cerebral infarction, or transient ischemic attacks), connective tissue diseases (rheumatoid arthritis, other arthritis, vasculitis (excluding aortitis), systemic lupus, myositis, systemic sclerosis, Sjögren syndrome, Behçet's disease, sarcoidosis), malignancies, peripheral vascular disease (atherosclerosis, arterial embolism, or thrombosis, Raynaud's syndrome, intermittent claudication, excluding aneurysm of the aorta), moderate to severe renal disease, infectious diseases (for infectious diseases, only infectious episodes within the five years before surgery were considered, whether caused by bacteria, viruses, or parasites). We also identified the main diagnoses related to the indication for the surgery (aneurysm and dissection, pathology of the aortic valve (mainly aortic valve insufficiency), malformation of the circulatory system (mainly bicuspid aortic valve), and infection of the valves). The ICD codes used for the study are provided in Additional File 1.

### Statistical analysis

We first determined the prevalence of patients undergoing resection of the ascending part of the aorta whose tissue was submitted for pathologic examination and the prevalence of aortic inflammation among those examined. We also examined the distribution of age groups, gender, presence of an aneurysm, aortic valve pathology, and the aortitis risk factors described above among patients with and without pathologic examination. Next, we compared pathologically examined patients with proof of aortitis with those without aortitis. We used logistic regression to compute adjusted prevalence odds ratios (ORs) for aortitis among persons with and without a given predictive factor, with associated 95% confidence intervals (CIs). Pre-defined predictive factors were: sex, age at surgery (categorized according to quartiles: 15 to 47 years, 48 to 59 years, 60 to 67 years, and 68 to 84 years), past history of hypertension, diabetes, stroke, ischemic heart disease, peripheral vascular disease, renal failure, connective tissue disease, infection, cancer, and surgical center. With data available on pathologic examinations in 600 surgical patients during the study period and with an expected aortitis prevalence rate of 5% based on the literature, we had 80% power to detect an OR of 3.0 for an aortitis risk factor with a prevalence of 15% in the study population.

In a second logistic regression model we examined predictors for performance of a pathologic examination as the outcome variable, in order to assess potential detection biases. Statistical analysis was performed using SAS software (version 9.1, SAS institute Inc., Cary, NC, USA).

The databases used in this study were accessible with permission from the Danish Data Protection Agency, and the study was approved by the Aarhus University Hospital Registry Board. According to Danish law, purely registry-based research that does not involve direct contact with the patients or biologic specimens does not require an additional permission from the patient.

## Results

Between 1997 and 2009, 1,210 patients over the age of 15 years underwent resection of the ascending portion of the aorta. Of these, 610 had a sample of tissue from the aorta submitted for pathologic examination (50.4%). Among patients with pathologic examination, 37 were diagnosed with inflammation of the aortic wall (6.1%). Of these patients, 10 were diagnosed with a condition known to be closely associated with aortitis or aortic aneurysm (5 with previously diagnosed temporal arteritis, 1 with Crohn's disease, 1 with rheumatoid arthritis, 1 with systemic lupus erythematosus, 1 with infectious aortitis, and 1 with Marfan's disease). Thus, 27 patients had idiopathic aortitis. Among the 37 patients with aortitis, granulomatous inflammation or presence of giant cells were reported in 8 patients. Aortitis patients were significantly older than those without this condition: their mean age was 65 (range: 57 to 70) years vs. 59 (range: 47 to 67) years for patients without aortitis (***P***= 0.03). Patients diagnosed with aortitis were predominantly men (62%), as were patients without aortitis (68.9%; ***P***= 0.39).

The main recorded indications for surgery are listed in Table 1. As expected, aortic aneurysm and dissection were the most common indications (76.2% of patients). Aortic valve insufficiency was coded in 74.5% of patients undergoing surgery. In logistic regression analyses, valve dysfunction was associated with aortitis (OR 2.3, 95% CI 1.1 to 4.9) when aneurysm/dissection was controlled for (Table 1). Bicuspid aortic valve was the most commonly reported malformation (40% of patients with a malformation of the circulatory system).

**Table 1:** Main indications for surgery of the ascending aorta in 1,210 patients

Variable^a^	With pathological examination of aorta				**Without pathological examination of aorta**, *n *= 600 (%)
	**Without aortitis, *n *= 573 (%)**	**With aortitis, *n *= 37 (%)**	**OR for aortitis (95% CI)**	***P***	

Aneurysm^b^	469 (82)	30 (81.1)	1.3 (0.5-3.3)	0.53	423 (81.8)
Pathology of the aortic valve^c^	261 (45.6)	24 (64.9)	2.3 (1.1-4.9)	0.027	279 (46.6)
Malformation^d^	21 (3.7)	1 (2.7)	0.7 (0.1-5.2)	0.69	19 (3.2)
Infection	8 (1.4)	1 (2.7)	1.7 (0.2-14.9)	0.61	30 (5)

Patients with and without pathological examination and with and without a diagnosis of aortitis, Denmark, 1997 to 2009 (logistic regression analysis, adjusted prevalence ORs for aortitis).

CI, confidence interval; OR, odds ratio.

^a ^Several conditions/indications could be present for the same patient.

^b ^With or without dissection.

^c ^Refers to aortic valve functional status, i.e. insufficiency, stenosis.

^d ^Refers to anatomic information, i.e., bicuspid valve, other congenital malformation.

The prevalence of potential risk factors for aortitis is summarized in Table 2 (logistic regression analysis, adjusted ORs). Aortitis patients were older than patients without inflammation, and the OR for aortitis among patients aged older than 67 years was 2.5 (95% CI 0.8 to 7.5). Among comorbidities, a history of connective tissue disease was a strong risk factor for aortitis (OR 4.7, 95% CI 1.6 to 13.6). Diabetes was associated with a markedly increased risk for aortitis (OR 5.2, 95% CI 0.9 to 29.7), although statistical precision was limited. Pathologies associated with atherosclerosis (ischemic heart disease, cerebrovascular disease, and peripheral vascular disease) were not associated with aortitis, corresponding to adjusted ORs close to one. Also, potential triggers in the pathophysiology of aortitis (such as past history of cancer and infection) did not prove to be risk factors for aortitis in our study.

**Table 2:** Demographics variables, comorbidities, and cardiovascular risk factors in 1,210 patients

Variable	With pathological examination of aorta				Without pathological examination of aorta, n (%)
	**Without aortitis, n (%)**	**With aortitis, n (%)**	**OR for aortitis (95% CI)**	**p**	

Male gender	395 (69)	23 (62)	1.1 (0.5-2.4)	0.78	410 (68)
Cancer	37 (6)	2 (5)	0.7 (0.1-3.2)	0.63	51 (8)
Stroke	56 (10)	3 (8)	0.8 (0.2-2.8)	0.72	95 (16)
Ischemic heart disease	163 (28)	10 (27)	0.8 (0.4-1.9)	0.70	227 (38)
Renal failure	36 (6)	1 (3)	0.5 (0.1-4.0)	0.51	49 (8)
Connective tissue disease	28 (5)	7 (19)	4.7 (1.6-13.6)	0.0042	33 (5)
Peripheral vascular disease	47 (8)	3 (8)	0.8 (0.2-2.9)	0.72	55 (9)
Infection	83 (14)	3 (8)	0.4 (0.1-1.5)	0.17	107 (18)
Hypertension	183 (32)	13 (35)	1.2 (0.5-2.5)	0.68	261 (43)
Diabetes	10 (2)	2 (5%	5.2 (0.9-29.7)	0.06	19 (3)
Age, years				0.35	
15-47	148 (26)	6 (16)	1.0 (ref.)		79 (13)
48-59	147 (26)	8 (21)	1.4 (0.5-4.4)		145 (24)
60-67	143 (25)	9 (24)	1.5 (0.4-4.5)		156 (26)
68-84	135 (2)	14 (38)	2.5 (0.8-7.6)		220 (37)
Surgical center				0.11	
1	118 (21)	9 (24)	1.0 (ref.)		238 (40)
2	110 (19)	2 (5)	0.2 (0.1-1.0)		54 (9)
3	114 (20)	5 (14)	0.6 (0.2-2.1)		55 (9)
4	162 (28)	13 (35)	1.1 (0.4-2.9)		219 (36)
5	69 (12)	8 (22)	1.7 (0.6-4.9)		34 (6)

Patients with and without pathological examination, and with and without inflammation of the ascending aorta, Denmark, 1997 to 2009 (logistic regression analysis, adjusted prevalence ORs for aortitis).

CI, confidence interval; OR, odds ratio.

The proportion of patients for whom a tissue sample was submitted for pathologic examination differed greatly by surgical center in Denmark, ranging from 35% to 69%. Interestingly, surgical departments that performed more aortic resections were less likely to send tissue samples to the pathologist. Factors associated with a pathologic examination were the center where the patient underwent surgery (OR 4.5, 95% CI 2.8 to 7.3 for examination at the center with most examinations vs. the reference center with least examinations) and aneurysm or dissection as the surgical indication (OR for pathologic examination 1.9, 95% CI 1.4 to 2.7; data not shown). A past history of hypertension (OR 0.7, 95% CI 0.6 to 0.9) and older age (OR 0.3, 95% CI 0.2 to 0.5) were negatively associated with a pathologic examination, as was a diagnosis of infection of the valve (OR 0.4, 95% CI 0.2 to 0.8).

## Discussion

In our nationwide population-based study, we found that 6.1% of patients undergoing resection of the ascending portion of the aorta in Denmark had pathologically proven inflammation of the aortic wall. Of these, most had idiopathic aortitis (73%) with no condition classically known to be closely associated with aortitis or aortic aneurysm. We found that a history of connective tissue disease was strongly associated with an increased risk of aortitis at the time of surgery, independent of other predictors examined. The fact that the rate of pathologic examination was similar in patients with and without connective tissue disease strengthens the credibility of this association. Advanced age tended to predict aortitis, as did a history of diabetes which was associated with a five times increased risk of aortitis.

The prevalence of aortitis among patients undergoing resection of the ascending portion of the aorta in Denmark (6.1%) is remarkably consistent with previous studies conducted at single medical centers: 4.3% at the Cleveland Clinic, Ohio, USA [28] (infectious aortitis patients were excluded), 8.7% at the Mayo Clinic, Minnesota, USA [16] (infectious aortitis patients also were excluded), and 4.9% at the Orsola-Malpighi Hospital, Bologna, Italy [29].

In Denmark, the prevalence of aortitis was similar in both sexes. In other reported series, women were predominantly affected (range: 61.5% to 82%) [28,29]. Although the median age of patients with aortitis in our study was 65 years, the mean age in previous studies ranged from 63 to 72 years [16,28,29].

In our study, prevalence of atherosclerotic diseases (ischemic heart disease, cerebrovascular disease, and peripheral arterial disease) was similar in patients with and without aortitis. This differs from previous research reporting that ascending thoracic aneurysms are associated with less systemic atherosclerosis [33] and that atherosclerotic profiles differ between patients with thoracic and abdominal aortic aneurysms [34]. In a case-control study of 50 idiopathic aortitis patients and 100 age-matched controls focusing on cardiovascular risk factors, Chowdhary et al. found that female gender (OR 2.4, 95% CI 1.2 to 4.8) and current smoking (OR 3.2, 95% CI 1.05 to 9.9) were associated with idiopathic aortitis [20], but not hypertension, hyperlipidemia, or diabetes mellitus. Also, smoking has been found to be strongly associated with giant cell arteritis in women [27]. Data on smoking status unfortunately were not available in our database, and we also had no data on lipid profile or family history.

The trigger for the inflammatory process underlying aortitis remains unknown. Specific activation of the adventitial dendritic cells of the arterial wall by pathogen-derived macromolecules is a critical event in the initiation of temporal arteritis [35,36], and this may provide clues for studying the pathophysiology of aortitis. Several studies have tried to identify potent infectious pathogens triggering temporal arteritis [37,38] and some case series have suggested a potential relation between vasculitis and cancer [23]. We thus tried to identify whether a history of cancer or infectious disease was associated with aortitis, but failed to find such an association.

Our study was restricted to the subgroup of aortitis patients with complications requiring a surgical procedure, and for whom a surgical sample was submitted for pathologic examination. Patients with aortitis not requiring surgical intervention or with asymptomatic mild disease thus were not included in our study. These limitations are shared in part by other studies on this topic. However, restricting our sample to patients with a pathologic sample allowed accurate diagnosis of the inflammation of the aortic wall and enhanced the study's internal validity. Another limitation of our database study is the lack of clinical detail concerning the inflammation, including acute phase reactants and imaging details.

Pathologic examination of the aorta was performed in only half of the patients undergoing surgery, reflecting usual practice in Denmark. The amount of tissue submitted for examination differed by center and the habits of individual surgeons. Pathologic examination occurred less frequently in patients with a diagnosis of endocarditis, perhaps because a tissue sample was submitted for bacterial culturing rather than for pathologic examination. History of hypertension and older age also were negatively associated with a pathologic examination. Although aortic aneurysms are more common in this population, a diagnosis of aortitis was not suspected before pathologic examination in published surgical series [17,29]. Thus gross inspection during surgery cannot replace pathologic examination. The prognosis may be worse for patients with aortitis than for patients with ordinary aortic aneurysms, and postoperative complications also may be more frequent [17,29]. This highlights the need for systematic pathologic examination of the aorta, if surgically feasible, even in daily practice involving an unselected population (older patients with classical risk factors for aortic aneurysm such as hypertension), and even if optimal treatment for active aortitis has yet to be defined.

Aortitis may be underdiagnosed for several reasons: the course of the disease may remain asymptomatic for a long time; patients are diagnosed when complications occur, mainly in the form of aortic aneurysms requiring surgery; and half of surgical samples are not submitted for pathologic examination, and some cases of idiopathic aortitis may not be recognized.

The distribution of potential cardiovascular risk factors was similar in patients with and without pathologic examination, which argues against potential detection bias in our study. However, patient age may have introduced bias. As a tissue sample is less frequently sent for pathologic examination in the case of elderly patients, our analysis of risk factors may have underestimated the association between older age and aortitis. This may at least partially explain why older age did not reach statistical significance as a risk factor in our model.

One of our study's main strengths is its nationwide population-based design. It is the first study to use a nationwide population-based cross-sectional design spanning 13 years and set in a country with more than five million residents. The uniform organization of health care in Denmark facilitated the study, as surgical procedures involving the aorta are performed in only five tax-supported hospitals in Denmark, with free access for patients. All procedures are recorded in the DNPR and the civil personal number permits accurate linkage between databases (i.e., DNPR and the National Pathology Registry). This allowed us to establish a complete hospitalization history for each patient. The availability of national registries also allowed us to collect exhaustive data on comorbidities such as diabetes, cancers, and infectious diseases, which could play a role in the pathogenesis of aortitis [39,40].

Our population-based design allowed us to determine the exact proportion of idiopathic vs. secondary aortitis of the ascending portion of the aorta among patients undergoing surgery, avoiding the potential selection biases that may occur in vasculitis referral centers. Idiopathic aortitis accounts for 75% of all aortitis cases, and is therefore the most common type of aortitis but the least examined until now.

## Conclusions

During the 1997 to 2009 period, pathologically confirmed aortitis was present in 6% of patients undergoing resection of the ascending part of the aorta in Denmark. This prevalence underscores the value of systematic pathologic examination of removed tissue. The majority of cases were classified as 'idiopathic', with known vasculitides or inflammatory conditions found only in 27% of cases. Idiopathic aortitis thus is a condition deserving further epidemiologic and pathophysiologic studies, with emphasis on older patients and patients with diabetes. Finally, it must be noted that the surgical procedure does not allow for assessment of the extension of the inflammatory process in the aortic arch. Thus the prognosis of patients with aortitis and the potential evolution of the inflammatory process in the remaining aorta should be assessed in future studies.

## Abbreviations

CI: confidence interval; DNPR: Danish National Patient Registry; ICD: International Classification of Diseases; NOMESCO: nordic medico-statistical committee; OR: odds ratio; SNOMED: systematized nomenclature of medicine.

## Competing interests

The authors declare that they have no competing interests.

## Authors' contributions

JS conceived and designed the study, analyzed the data, performed the statistical analysis, and wrote the draft manuscript. KS and JK participated in designing the study, and in analyzing the data. PD participated in drafting the manuscript. RT participated in conceiving and designing the study, analyzing the data, and drafting the manuscript. All authors read and approved the final manuscript.

## Supplementary Material

Additional file 1 **Primary diagnoses associated with surgery of the ascending aorta and International Classification of Diseases (ICD)-8 and ICD-10 codes used to identify comorbidities**. Click here for file
