# Supplementary material for: Predictors for pathologically confirmed aortitis after resection of the ascending aorta: A 12-year Danish nationwide population-based cross-sectional study
Source: Arthritis Res Ther. 2011 Jun 15;13(3):R87. doi: 10.1186/ar3360 (PMC3218902; doi:10.1186/ar3360)
Supplement: Additional file 1 — Primary diagnoses associated with surgery of the ascending aorta and International Classification of Diseases (ICD)-8 and ICD-10 codes used to identify comorbidities. [file ar3360-S1.DOC]

**Appendix 1:** Primary diagnoses associated with surgery of the ascending aorta and International Classification of Diseases (ICD)-8 and ICD-10 codes used to identify comorbidities.

| Diagnosis | ICD-8 codes | ICD-10 codes |
| --- | --- | --- |
|  |  |  |
| Aneurysm +/- dissection |  | I71 |
| Pathology of the aortic valve |  | I35, I06 |
| Malformation of the circulatory system |  | Q2 |
| Endocarditis |  | I33, I38, I39 |
| Cancer | 14, 15, 16, 17, 18, 190, 191, 192, 193, 194, 204, 205, 206, 207, 200, 201, 202, 203, 27559, 195, 196, 197, 198, 199 | C0, C1, C2, C3, C4, C5, C6, C70, C71, C72, C73, C74, C75, C91, C92, C93, C94, C95, C81, C82, C83, C84, C85, C88, C90, C96, C76, C77, C78, C79, C80 |
| Stroke | 431, 432, 433, 434, 435, 436, 437, 438, 344 | I61, I63, I64, I65, I66, G45, G81, G82 |
| Ischemic heart disease | 410, 411, 412, 413 | I20, I21, I22, I24, I25 |
| Renal failure | 403, 404, 580, 581, 582, 583, 584, 59009, 59319, 7531, 792 | I12, I13, N00, N01, N02, N03, N05, N07, N11, N14, N17, N18, N19, Q61 |
| Connective tissue disease | 712, 716, 734, 446, 13599 | M05, M06, M08, M09, M30, M31, M32, M33, M34, M35, M36, D86 |
| Pulmonary disease | 490, 491, 492, 493, 515, 516, 517, 518 | J40, J41, J42, J43, J44, J45, J46, J47, J60, J61, J62, J63, J64, J65, J6, J67, J684, J701, J703, J841, J920, 961, J982, J983 |
| Peripheral vascular disease | 440, 442, 443, 444, 445 | I70, I72, I73, I74, I77 |
| Ulcer | 53091, 53098, 531, 532, 533, 534 | K221, K25, K26, K27, K28 |
| Infection | 0, 10, 11, 12, 13, 320, 322, 382, 383, 42000 421, 461, 463, 470, 471, 472, 473, 474, 48, 501, 503, 510, 513, 540, 590, 601, 612, 620, 622, 680, 681, 682, 683, 684, 685, 686, 710, 720 | A, B, G00, G01, G02, G05, G06, G07, H70, I301, I320, I33, I38, I39, I400, I410, I410, I412, J09, J10, J11, J12, J13, J14, J15, J16, J17, J18, J20, J21, J22, J85, J86, L0, M00, M01, M02, M03, N10, N41, N70, N71, N72, N73, N74, N75, N76, N77, J32, J36 |
| Hypertension | 400, 401, 402, 403, 404 | I10, I11, I12, I13, I15 |
| Diabetes | 249, 250 | E10, E11, E12, E13, E14 |
